# Supplementary material for: Ciliated Cells in Ovarian Cancer Decrease with Increasing Tumor Grade and Disease Progression
Source: Cells. 2022 Dec 11;11(24):4009. doi: 10.3390/cells11244009 (PMC9776429; doi:10.3390/cells11244009)
Supplement: Supplementary file 1 [file cells-11-04009-s001.zip › cells-2020219-supplementary.pdf]

A

NORMAL FALLOPIAN TUBE AND ENDOMETRIUM

% CAPS+ cells/case	Tissue	Stage	Age	TMA
50	Fallopian tube	-	61	2
40	Fallopian tube	-	67	2
40	Fallopian tube	-	61	2
40	Fallopian tube	-	64	2
30	Endometrium	-	57	2
20	Endometrium	-	43	2
20	Endometrium	-	43	2
20	Endometrium	-	46	2
20	Endometrium	-	41	2
20	Endometrium	-	57	2

BENIGN CYSTADENOMA

% CAPS+ cells/case	Tissue	Stage	Age	TMA
70	Serous	-	76	2
50	Serous	-	59	1
50	Serous	-	74	2
40	Serous	-	71	2
40	Serous	-	46	2
30	Serous	-	36	1
0	Serous	-	53	2
20	Mucinous	-	29	1
0	Mucinous	-	42	1
0	Mucinous	-	48	1
0	Mucinous	-	51	2
0	Mucinous	-	42	2
0	Mucinous	-	60	2
0	Mucinous	-	76	2
0	Mucinous	-	42	2
0	Mucinous	-	25	2

BORDERLINE TUMOR

% CAPS+ cells/case	Tissue	Stage	Age	TMA
70	Serous	-	46	2
60	Serous	-	49	2
50	Serous	-	39	2
50	Serous	-	44	3
40	Serous	-	65	1
40	Serous	-	65	3
30	Serous	-	43	1
20	Serous	-	44	1
20	Serous	-	55	1
20	Serous	-	35	3
10	Serous	-	46	2
0	Serous	-	57	2
0	Serous	-	72	2
0	Mucinous	-	25	1
0	Mucinous	-	25	1
0	Mucinous	-	35	1
0	Mucinous	-	38	1
0	Mucinous	-	21	1
0	Mucinous	-	70	2
0	Mucinous	-	50	2
0	Mucinous	-	13	2
0	Mucinous	-	67	2
0	Mucinous	-	54	2
0	Mucinous	-	49	2
0	Mucinous	-	26	3

LOW-GRADE CARCINIOMA

% CAPS+ cells/case	Tissue	Stage	Age	TMA
60	Serous	IIB	43	1
50	Serous	IIB	43	1
10	Serous	I	57	1
10	Serous	IIIA	51	1
1	Serous	IA	26	1
1	Serous	II	63	1
0	Serous	IA	56	1
70	Endometrioid	IA	67	2
70	Endometrioid	IC	81	4
50	Endometrioid	IA	22	4
50	Endometrioid	IB	58	4
50	Endometrioid	IC	51	2
50	Endometrioid	IC	47	4
40	Endometrioid	IA	72	4
10	Endometrioid	IC	52	2
0	Endometrioid	IC	83	2
0	Mucinous	IA	20	2
0	Mucinous	IA	43	2
0	Mucinous	IA	63	2
0	Mucinous	IA	50	4
0	Mucinous	IC	61	4

B

HIGH-GRADE CARCINOMA

% CAPS+ cells/case	Tissue	Stage	Age	TMA
50	Serous	IIIC	64	3
30	Serous	IIC	45	3
20	Serous	IIA	42	2
10	Serous	IA	40	3
1	Serous	I	51	1
1	Serous	I	52	1
1	Serous	II	36	1
1	Serous	II	66	1
1	Serous	IIC	50	3
1	Serous	III	75	1
1	Serous	IIIA	49	1
1	Serous	IIIA	43	3
1	Serous	IIIB	71	2
1	Serous	IIIB	70	3
1	Serous	IIIC	62	2
1	Serous	IIIC	65	2
1	Serous	IIIC	66	2
1	Serous	IIIC	51	3
0	Serous	I	46	1
0	Serous	I	50	1
0	Serous	I	63	1
0	Serous	IA	47	1
0	Serous	IA	51	1
0	Serous	IA	54	1
0	Serous	IA	56	1
0	Serous	IB	70	1
0	Serous	IIA	64	2
0	Serous	IIB	62	1
0	Serous	IIB	58	3
0	Serous	IIB	65	3
0	Serous	IIB	57	3
0	Serous	III	52	1
0	Serous	IIIA	50	1
0	Serous	IIIA	69	1
0	Serous	IIIA	48	1
0	Serous	IIIB	64	2
0	Serous	IIIB	64	2
0	Serous	IIIB	43	3
0	Serous	IIIB	55	3
0	Serous	IIIC	64	1
0	Serous	IIIC	62	2
0	Serous	IIIC	74	2
0	Serous	IIIC	45	2
0	Serous	IIIC	52	2
0	Serous	IV	52	1
0	Serous	IV	50	3
20	Mucinous	IC	47	4
0	Mucinous	IA	52	1
0	Mucinous	IA	25	1
0	Mucinous	IA	63	2
0	Mucinous	IA	57	2
0	Mucinous	IA	40	2
0	Mucinous	IC	69	4
0	Mucinous	IC	47	4
0	Mucinous	IA	74	4
0	Mucinous	IIB	75	1
0	Mucinous	IIB	48	1
0	Mucinous	IIB	29	2
0	Mucinous	IIC	63	4
40	Endometrioid	IC	68	1
40	Endometrioid	III	48	4
30	Endometrioid	IIB	52	2
10	Endometrioid	IB	34	1
1	Endometrioid	IC	45	3
1	Endometrioid	IIB	37	3
1	Endometrioid	IIB	64	4
1	Endometrioid	IIIC	41	4
1	Endometrioid	IV	47	4
0	Endometrioid	1A	65	1
0	Endometrioid	1A	49	1
0	Endometrioid	IA	47	1
0	Endometrioid	IA	62	1
0	Endometrioid	IA	82	1
0	Endometrioid	IB	51	1
0	Endometrioid	IC	45	1
0	Endometrioid	IC	50	1
0	Endometrioid	IC	54	2
0	Endometrioid	IC	53	2
0	Endometrioid	IC	81	2
0	Endometrioid	II	68	3
0	Endometrioid	IIB	48	3
0	Endometrioid	IIB	49	4
0	Endometrioid	IIIA	53	4
0	Endometrioid	IIIB	45	4
0	Endometrioid	IIIC	62	4
0	Endometrioid	IIIC	51	4
0	Endometrioid	IIIC	42	4
0	Endometrioid	IV	32	4
50	Clear cell	IA	41	3
20	Clear cell	IIC	31	4
20	Clear cell	IIIC	56	4
1	Clear cell	IA	58	3
1	Clear cell	IC	64	4
1	Clear cell	IC	60	4
1	Clear cell	IIB	53	4
1	Clear cell	IIC	59	4
1	Clear cell	IIIC	78	4
0	Clear cell	IA	72	2
0	Clear cell	IA	39	2
0	Clear cell	IA	50	3
0	Clear cell	IC	62	2
0	Clear cell	IC	88	2
0	Clear cell	IC	50	2
0	Clear cell	IC	52	4
0	Clear cell	IIB	41	3
0	Clear cell	IIC	73	2
0	Clear cell	IIC	76	2
0	Clear cell	IIC	47	4
0	Clear cell	IIC	66	4
0	Clear cell	IIC	61	4
0	Clear cell	IIIA	77	2
0	Clear cell	IIIA	42	2
0	Clear cell	IIIA	68	2
0	Clear cell	IIIC	27	2
0	Clear cell	IIIC	39	3
0	Clear cell	IIIC	50	4
0	Clear cell	IIIC	82	4
0	Clear cell	IVB	38	3

Table S1. Detection and quantitation of CAPS+ cells in TMAs 1-4. (A) Normal fallopian tubes and endometria, ovarian benign cystadenomas, borderline tumors, and low-grade carcinomas. (B) Ovarian high-grade carcinomas.
